# Direct coupling of size exclusion chromatography and mass spectrometry for the characterization of complex monoclonal antibody products

**DOI:** 10.1002/jssc.202200075

**Published:** 2022-03-28

**Authors:** Amarande Murisier, Marie Andrie, Szabolcs Fekete, Matthew Lauber, Valentina D'Atri, Katharina Iwan, Davy Guillarme

**Affiliations:** ^1^ Institute of Pharmaceutical Sciences of Western Switzerland (ISPSO) University of Geneva Geneva Switzerland; ^2^ School of Pharmaceutical Sciences University of Geneva Geneva Switzerland; ^3^ Waters Corporation Milford Massachusetts USA; ^4^ Roche Diagnostics GmbH Penzberg Germany

**Keywords:** bioinert columns, low adsorption column hardware, mass spectrometry, monoclonal antibodies, size‐exclusion chromatography

## Abstract

The present study describes the possibilities offered by an innovative bioinert size exclusion chromatography column for size variant characterization of complex monoclonal antibody products. This size exclusion chromatography column includes a novel column hardware surface. The column was prepared from metallic hardware components that were treated to have prototype hydrophilically modified hybrid organic–inorganic silica surfaces called hybrid surface technology. This provides a significant reduction in nondesired hydrophobic and electrostatic interactions that can occur between column and analyte when performing size exclusion chromatography analysis with volatile mobile phase.

Compared to a reference stainless‐steel column packed with the same batch of packing material, peak tailing, band broadening, and above all recovery of high molecular weight species were distinctly improved for all types of monoclonal antibody products. Based on our observations, we found that 50 mM ammonium acetate in water was a suitable mobile phase offering good compromise in terms of liquid chromatography performance and mass spectrometry sensitivity. In addition, method repeatability (intra‐ and interday relative standard deviations) on elution times and high molecular weight species peak areas were found to be excellent.

By using this innovative size exclusion chromatography material, the low and high molecular weight species contained in various stressed and nonstressed monoclonal antibody products were successfully characterized with mass spectrometry detection.

**Article Related Abbreviations**
ADC: antibody–drug conjugate
h‐HST: hydrophilically modified HST
HMW: high molecular weight species
HST: hybrid surface technology
LMW: low molecular weight species
PEEK: polyether ether ketone
SEC: size exclusion chromatography
SS: stainless steel

## 1 INTRODUCTION

Size exclusion chromatography (SEC) is a reference technique for the qualitative and quantitative analysis of protein size variants, including fragments and aggregates [1]. SEC is considered to be a nondenaturing technique, since mild conditions (aqueous mobile phase, ambient temperature, and low pressure) are applied, enabling the characterization of biomolecules without affecting their structure, conformation, or local environment [2]. Ideally, SEC separations are entropically controlled processes (with zero enthalpy term, *ΔH* = 0). Therefore, packing materials should be chemically inert to minimize any adsorptive interactions. The separation (selectivity) is exclusively based on the differences between residence times spent in the internal pores of the SEC media, which itself is determined by internal pore diameter and pore size distribution [3]. Elution times and selectivity are accordingly related to the hydrodynamic radii of the solutes. The high molecular weight species (HMWs) elute first, followed by the monomer and finally the fragments (truncations) or other types of low molecular weight species (LMWs).

In many cases, the ideal conditions are not met, and nonspecific (nondesired) adsorption (due to hydrophobic and electrostatic interactions) may occur on either the packing material or even the surface of the column hardware. Those interactions lead to irreversible protein adsorption (low recovery), shifted elution time, peak tailing, and band broadening. To limit those nondesired interactions with the packing material, mobile phase additives like high ionic strength buffers, high concentrations of counter ions, arginine, or the addition of low amounts of organic modifiers are often used in an attempt to improve aggregate recoveries and peak shapes [4, 5, 6, 7, 8, 9].

In addition to redesigning the chemical properties of the packing material, it is possible to improve aggregate recovery and peak shape by making the column hardware less reactive. Stainless steel (SS) hardware components (column frits and inner column walls) have a propensity to adsorb proteins. As a possibility, titanium column hardware has been shown to improve aggregate recovery for monoclonal antibody (mAb) samples compared to SS hardware [10]. Another alternative is to use polyether ether ketone (PEEK) lined column hardware for RP and hydrophilic interaction chromatography analysis to improve recovery of challenging samples [11]. Very recently, a new generation of PEEK lined (metal‐free) SEC column technology was applied to direct SEC‐MS analysis of protein biopharmaceuticals using volatile (ammonium acetate) mobile phase [12]. With such metal‐free column hardware, significantly improved aggregate recovery was observed for several mAbs and antibody–drug conjugate (ADC) samples when compared to its SS counterpart. As expected, the largest differences between SS and PEEK lined hardware were observed for the most basic mAbs (high p*I*), confirming the existence of electrostatic interactions between basic proteins and SS hardware. The issue of using volatile mobile phase additives for SEC‐MS analysis was also demonstrated in an earlier study, as low aggregate recovery was observed with ammonium acetate buffer compared to a commonly used phosphate buffer [13]. Only acidic proteins could be analyzed without significant loss of aggregate recovery when using volatile mobile phase, which is mainly due to hardware interactions.

Recently, hybrid organic−inorganic surfaces (hybrid surface technology [HST]) were introduced as column hardware material that can be used to decrease nondesired electrostatic interactions between column and analyte [14]. This surface is composed of an ethylene bridged siloxane polymer that is formed on metal substrates using a vapor deposition process. Besides the significant decrease in nonspecific ionic interactions, this surface was predicted to possess low hydrophobicity too. Contact angle measurements indicated that the HST is indeed more hydrophilic than PEEK, making it less prone to nondesired hydrophobic adsorption [15]. This HST surface was successfully applied in RP and hydrophilic interaction chromatography modes; however, it has not yet been applied to SEC [14, 16, 17]. When analyzing mAbs and ADCs possessing large hydrophobic surface areas or hydrophobic drugs, nondesired hydrophobic interactions can drastically limit the detection of aggregates in SEC.

The aim of this work was to study the possibilities of a new hydrophilically modified HST (h‐HST) surface in SEC when analyzing challenging proteins with volatile mobile phase (ammonium acetate). Custom columns of 150 × 4.6 mm, made up of XBridge Protein BEH SEC 200 Å, 2.5 μm material were used, where an additional hydrophilic layer was developed on the surface of the HST material and applied to column frits and internal column walls to decrease both electrostatic and hydrophobic interactions as much as possible. In this work, two types of column hardware were tested, namely conventional SS and hydrophilically modified h‐HST material. The columns were packed with the same batch of packing material and systematically compared using nonvolatile phosphate and volatile ammonium acetate mobile phases.

## 2 MATERIALS AND METHODS

### 2.1 Chemicals and reagents

Type 1 water was provided by a Milli‐Q purification system from Millipore (Burlington, MA, USA). Potassium phosphate dibasic (purity ≥ 99%), potassium phosphate monobasic (purity ≥ 99%), potassium chloride (KCl) as well as ammonium acetate (Ph.Eur ≥ 98%) and ammonium acetate solution (BioUltra, for molecular biology, ∼5 M) were obtained from Sigma Aldrich (Buchs, Switzerland).

### 2.2 Sample preparation

Therapeutic IgG monoclonal antibodies (∼145 kDa) including eculizumab and pembrolizumab and ADCs (∼150 kDa) brentuximab vedotin were obtained as European Union pharmaceutical‐grade drug products from their respective manufacturers. Additionally, ADC1 and mAbs1 were provided by Roche (Penzberg, Germany). Other mAb‐related complex samples including Fab (∼50 kDa), mAb‐cytokine‐fusion (∼165 kDa), mAb‐domain‐fusion (N‐terminal), and mAb‐domain‐fusion (C‐terminal) (∼200 kDa) were also provided by Roche and abbreviated bsAb1, bsAb2, bsAb3, and bsAb 4, respectively. Thermally stressed samples were obtained by incubation at 40°C during several weeks. Cartoon representations of sample structures are reported in Figure S1. Samples were diluted to 1 mg/mL with Milli‐Q water for SEC experiments and to 10 mg/mL for SEC‐MS experiments.

### 2.3 SEC experiments: Instrumentation and chromatographic conditions

SEC‐UV experiments were performed on a Waters ACQUITY UPLC I‐Class instrument (Waters, Milford, MA, USA), equipped with a Binary Solvent Manager, a flow‐through‐needle (FTN) injector, and a TUV detector equipped with a 5 mm long titanium cell of 1500 nL volume operating at 280 nm. Waters ACQUITY UPLC H‐Class Bio and Waters ACQUITY PREMIER were also used for the repeatability study and also include the same titanium UV cell. Custom columns of 150 × 4.6 mm, made of XBridge Protein BEH™ SEC 200 Å, 2.5 μm material made of conventional SS, and h‐HTS material were provided by Waters. SEC reference mobile phase consisted of 50 mM potassium phosphate buffer mixed with 250 mM KCl in MilliQ water, pH 6.8. Mobile phases consisting of volatile buffer made with 20, 50  and 100 mM ammonium acetate were also prepared. The mobile phases were systematically filtered through a 0.22 μm polyethersulfone membrane filter provided by Millipore. The flow rate was 0.4 mL/min and the injection volume was 5 μL.

The evaluation of injection repeatability across the same day, that is, intraday variability, was evaluated through measurements of aggregate recovery and elution time over a short period of time. The same samples and parameters were also evaluated over 3 days to determine the interday variability and finally the same experiments were conducted on different instruments (namely Waters ACQUITY UPLC I‐Class, Waters ACQUITY UPLC H‐Class Bio, and Waters ACQUITY PREMIER instruments) to obtain data of intra‐ and inter‐instrument variability.

The control of LC instruments for SEC‐UV experiments as well as data analysis was performed by Empower Pro 3 (Waters). Data processing was performed in Microsoft Excel.

### 2.4 SEC‐MS analyses: Instrumentation and experimental conditions

A UHPLC system (ACQUITY UPLC H‐Class, Waters, Milford, USA), equipped with a quaternary solvent delivery pump, an auto‐sampler including a 15‐μL flow‐through‐needle injector, and a TUV detector operating at 280 nm, was coupled to an ESI‐TWIMS‐Q‐TOF mass spectrometer (Vion, Waters, Milford, USA) to perform SEC‐MS analyses. The Vion was equipped with a narrow bore ESI probe enabling the use of flow rates lower than 0.1 mL/min. The instrument was operated in the sensitivity mode and positive polarity to acquire continuum data in the range of 1000–16,000 *m*/*z* with a scan time of 2 s. Capillary voltage was set at 3.0 kV, cone voltage at 150 V, and source offset at 80 V. Source temperature was set at 100°C, desolvation temperature at 500°C, and desolvation gas flow at 600 L/h. The system was calibrated by using a 200 pg/μL sodium iodide solution diluted in a mixture of water/isopropanol 50/50 (v/v) with 0.1% FA.

SEC‐MS analysis was performed with a prototype 150 × 4.6 mm, XBridge Protein BEH SEC 200 Å, 2.5 μm column made of h‐HTS material kept at room temperature. The separation was carried out in isocratic mode by using 50 mM ammonium acetate as mobile phase, a flow rate of 0.05 mL/min, and injection volume of 10 μL.

UNIFI v1.9.4 was used for data acquisition while protein mass spectra data treatment was performed with MassLynx software (Waters).

## 3 RESULTS AND DISCUSSION

### 3.1 Evaluation of h‐HST technology in SEC

It is expected that the recently developed HST material, based solely on an ethylene bridge hybrid siloxane composition, might mitigate strong electrostatic interactions, but that there might still be challenges in applying this chromatographic hardware to the separation of strongly hydrophobic biomolecules under aqueous conditions [14]. Thus, the ethylene‐bridged hybrid surface was further modified through the incorporation of a hydrophilic surface layer. This hydrophilically modified analog of the ethylene‐bridged surface (h‐HST) is expected to significantly reduce both nonspecific electrostatic and hydrophobic interactions between column hardware and solutes. As a result, improvements in aggregate recovery and peak shape are expected if it were to be applied to SEC conditions [18].

**FIGURE 1:**
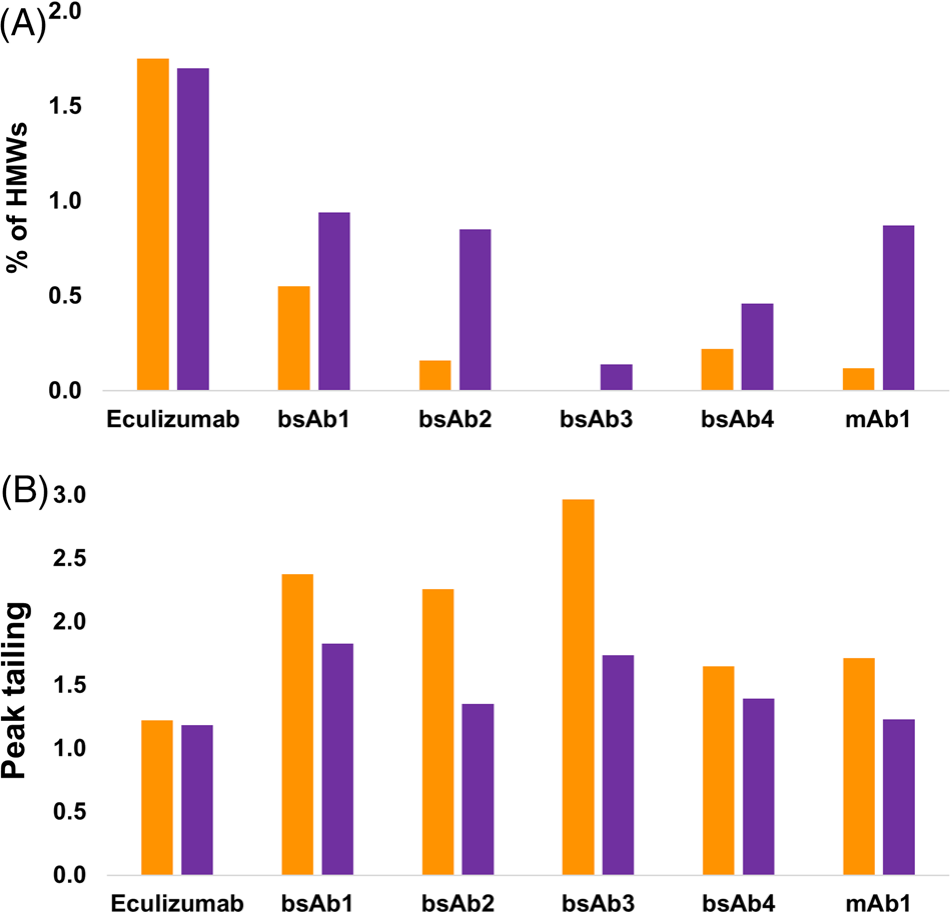
Comparison of %HMWs (A) and peak tailing (B) observed for various mAbs and mAb‐related products under size exclusion chromatography (SEC) conditions with volatile mobile phase. The orange and purple bars correspond to the reference SS BEH200 SEC and prototype h‐HST BEH200 SEC columns, respectively. Mobile phase: ammonium acetate 100 mM in water

**FIGURE 2:**
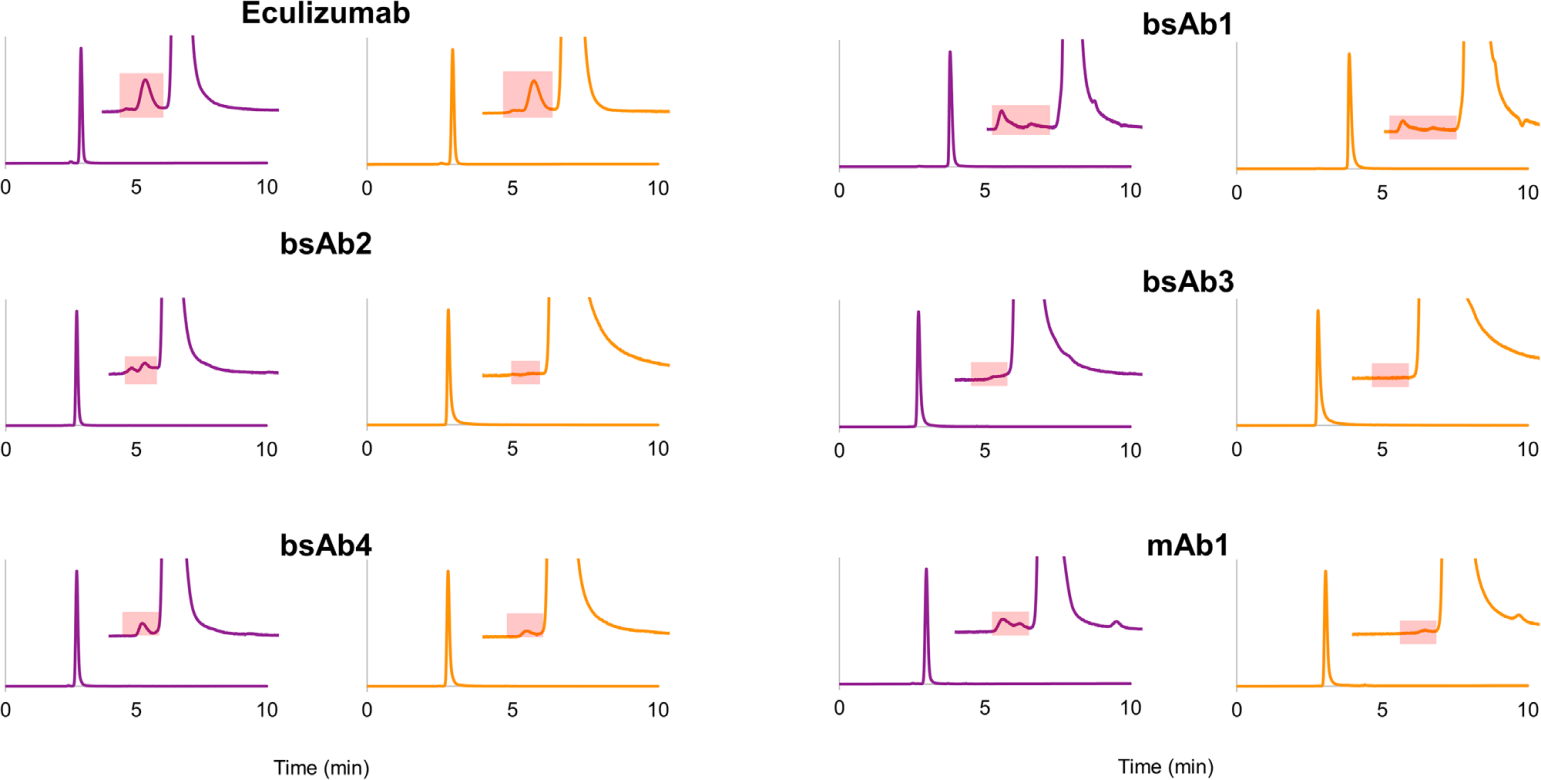
Chromatograms obtained with various mAbs and mAb‐related products under size exclusion chromatography (SEC) conditions with volatile mobile phase. The orange and purple chromatograms correspond to the reference SS BEH200 SEC and prototype h‐HST BEH200 SEC columns, respectively. The red boxes correspond to the zone where the high molecular weight species (HMWs) are eluted

First, aggregate recovery (area% of HMWs) of the four research samples (bsAb1, bsAb2, bsAb3, and bsAb4) plus two reference mAbs—eculizumab (acidic mAb, p*I* = 6.1) and mAb1 (strongly basic, p*I* = 9.4)—were systematically compared between the SS and h‐HST columns when using an aqueous mobile phase composed of a 100 mM ammonium acetate solution [19]. Figure 1A shows the obtained HMWs% recoveries, whereas Figure 2 shows the corresponding chromatograms.

For the reference mAbs, the acidic eculizumab showed very similar HMWs% with the SS and h‐HST hardware (1.75 and 1.70%, respectively), while the basic mAb1 showed only 0.12% HMWs with the SS hardware, but 0.87% (seven times more) with the h‐HST hardware. MAb1, but not eculizumab, is known to be an inordinately hydrophobic mAb [13]. Therefore, mAb1 can be considered a challenging sample. It is reported that this mAb is slightly retained in common SEC conditions and that its aggregates are underestimated with low ionic strength mobile phases [13, 20]. The much higher aggregate recovery for mAb1 observed with the h‐HST hardware suggests that indeed this new column hardware surface significantly decreases nondesired aggregate adsorption and probably gives a more reliable (accurate) estimation of HMWs%.

For all the four research samples, the h‐HST column hardware resulted in significantly higher aggregate recovery. For bsAb1, 1.7 times more aggregates (0.94 vs. 0.55% HMWs) were observed with the h‐HST hardware. The bsAb2 sample showed more than five times more aggregate, while the bsAb4 showed two times more aggregate with the h‐HST hardware versus SS. For the bsAb3 sample, no HMWs were detected with the SS column while 0.14% HMWs was observed with the h‐HST column.

Figure 1B shows the comparison of peak tailing factors obtained with the SS and h‐HST column hardware. When comparing the peak tailing (*T*_USP_) of the monomer species, a very similar conclusion can be drawn. The SS and h‐HST column hardware showed similar tailing only for eculizumab, otherwise the h‐HST hardware produced significantly less tailing for all other samples. These observations suggest that eculizumab probably does not tend to form nonspecific interactions. However, the other analytes, which are probably more basic and hydrophobic, form secondary interactions with the SS column hardware under the studied conditions. In conclusion, the new h‐HST hardware clearly mitigates a significant degree of nondesired interactions and improves aggregate recovery to the point of yielding more reliable analytical results.

### 3.2 Comparison of various mobile phase conditions

In the second step, we were interested in comparing different mobile phases including a reference 50 mM phosphate buffer and various ammonium acetate solutions (20, 50, and 100 mM). For this comparison, bsAb1, bsAb4, mAb1, and a strongly hydrophobic ADC (brentuximab vedotin) were considered. As the most promising column (see previous section), the h‐HST BEH200 column was selected.

**FIGURE 3:**
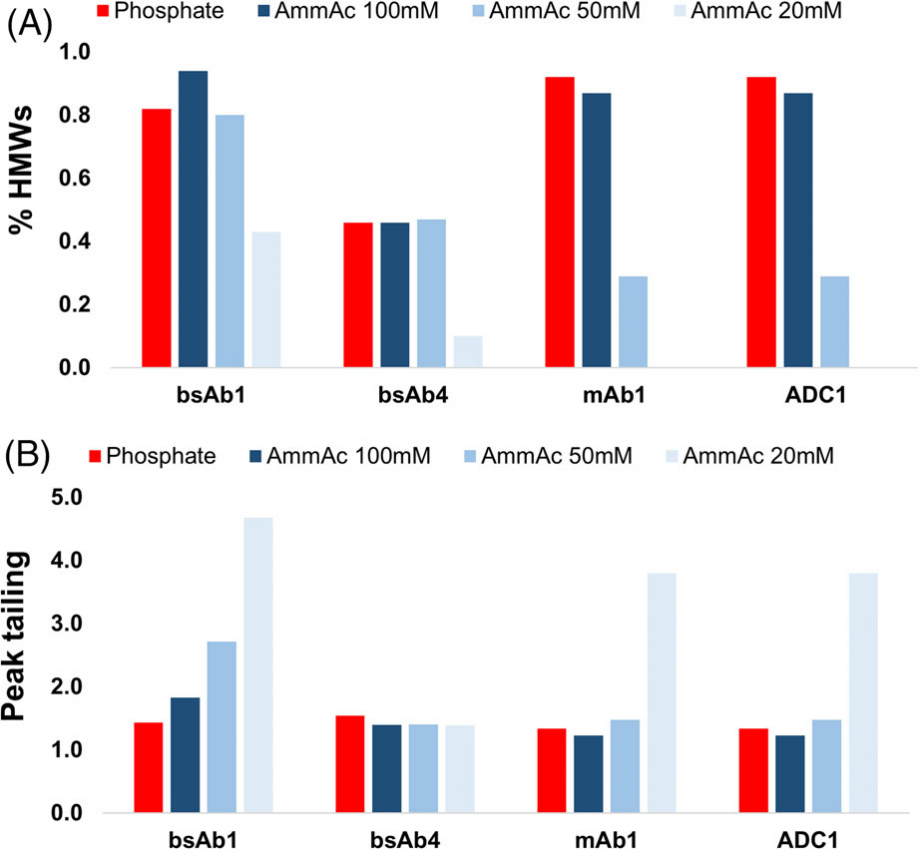
Comparison of %HMWs (A) and peak tailing (B) observed for various mobile phase conditions on the prototype h‐HST BEH200 SEC column. Mobile phase: 50 mM potassium phosphate + 250 mM KCl (reference) or ammonium acetate 20, 50, and 100 mM in water

For the bsAb4 sample, the HMWs recovery was very similar with 50 mM phosphate, 100 mM ammonium acetate, and 50 mM ammonium acetate (0.46, 0.46, and 0.47%, respectively), but a much lower amount of aggregates was detected with 20 mM ammonium acetate (only 0.1%). With the BsAb1 sample, 0.82, 0.94, 0.80, and 0.43% HMWs were obtained with the 50 mM phosphate and 100, 50, and 20 mM ammonium acetate mobile phases, respectively. The same behavior was observed with brentuximab vedotin, with comparable %HMWs being determined (i.e., 1.19 and 1.5) when using the 50 mM phosphate and 100 or 50 mM ammonium acetate mobile phases. A significant drop down to 0.54% was observed with 20 mM ammonium acetate. For mAb1, very similar ratios of HMWs were observed with 50 mM phosphate and 100 mM acetate buffers. A slightly lower amount was obtained with 50 mM acetate, and no HMWs were detected with 20 mM acetate. Figure 3A shows the corresponding plots of %HMWs as observed with the different mobile phases.

It was also of interest to compare peak tailing, which is another indicator of secondary interactions. For the bsAb4 and brentuximab vedotin samples, peak tailing was acceptable with all mobile phases (*T*_USP_ ranged between 1.39 and 1.54 for the bsAb4, while it is comprised between 1.40 and 1.75 for brentuximab vedotin). With bsAb1, peak tailing was only acceptable with the 50 mM phosphate and 100 mM ammonium acetate buffers, but the 50 and 20 mM ammonium acetate resulted in peak tailing *T*_USP_ > 2. MAb1 eluted in relatively symmetrical peaks T_USP_ < 1.5 with the reference buffer as well as the 100 and 50 mM ammonium acetate mobile phases. The 20 mM ammonium acetate mobile phase resulted in distorted peak shapes. Figure 3B shows these tailing factors plotted as a function of the different mobile phases.

Based on our observations, we found that 50 mM ammonium acetate is a good compromise for MS detection. Applying 50 mM ammonium acetate in combination with the h‐HST BEH200 column, peak tailing and HMWs recovery are acceptable, and MS compatibility is likewise improved. For comparison purpose, Figure S2 shows representative chromatograms for the 50 mM phosphate buffer and 50 mM ammonium acetate separation conditions.

### 3.3 Evaluation of method variability in SEC with volatile buffer

Next to the assessment of the most suitable column technology and mobile phase conditions for SEC‐MS operation, we have also evaluated whether the selected conditions were appropriate to obtain reliable %HMWs and consistent elution times. For this purpose, three representative samples were considered, namely mAb (pembrolizumab), ADC1, and bsAb4, which contain an average %HMWs equal to 1.01, 1.11, and 0.33%. These different samples were first injected on the reference UHPLC system (Waters Acquity UPLC I‐class equipped with Titanium UV cell) three times each day for 3 days. This allows for a calculation of intra‐ and interday variabilities on HMWs% and elution times expressed as RSDs (%RSD). In addition, three replicate injections were also performed on three different modern UHPLC instruments from the same manufacturer all equipped with a titanium UV cell, namely Waters Acquity UPLC I‐class, Waters Acquity UPLC H‐class Bio, and Waters Acquity Premier. This allowed for an assessment of intra‐ and interinstrument RSD values.

**FIGURE 4:**
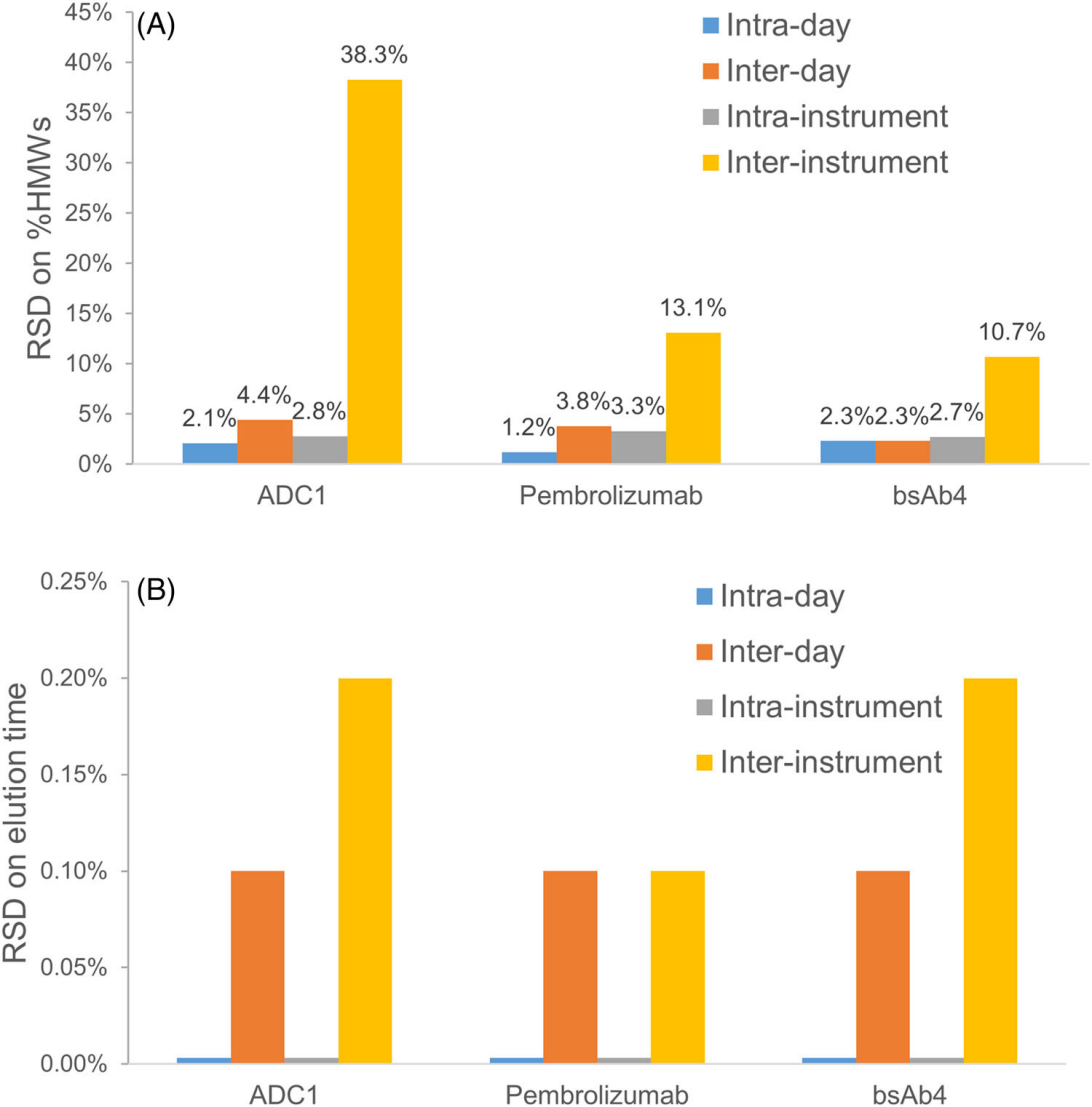
Comparison of RSD on %HMWs (A) and elution time (B) observed for various mAb and mAb‐related products on the prototype h‐HST BEH200 SEC column. Intra‐ and interday, and intra‐ and interinstrument RSDs were reported. Mobile phase: 50 mM ammonium acetate in water, titanium UV cell. Three different UHPLC instruments from Waters were employed for the intra‐ and interinstrument measurements: Acquity UPLC I‐Class, Acquity UPLC H‐Class Bio, and Acquity UPLC Premier

Figure 4A reports the RSD values for %HMWs of the three different samples. First, it is important to mention that the intraday RSD was excellent with values ranging from 1.2 to 2.3% depending on the analyzed sample. The interday RSD values were slightly larger, with values ranging from 2.3 to 4.4%. Surprisingly, the results obtained on the bsAb sample (the one containing the lowest amount of HMWs, 0.33% on average) were not worse than the ones obtained on the other two samples (both 1.1% on average). These results prove that an SEC method using volatile salts has the potential to be validated, even now with the use of 50 mM ammonium acetate. Next, we evaluated the effect of instrumentation on %HMWS. Intraday RSD values were comparable (comprised between 2.7 and 3.3%) no matter the employed instrument (1.2–2.3%). This means that any UHPLC instrument provides suitable repeatability. However, more significant differences were observed when considering all the values (three replicates on three different instruments), independently on the instrument. In this case, the RSD values were equal to 10.7% for the bsAb, 13.1% for the mAb, and up to 38.3% for the ADC product. This result confirms that some differences exist between the three instruments in terms of HMWs recovery, in particular for the most critical sample (ADC1, which is the most hydrophobic sample). Based on these results, it is clearly recommended to preferentially use bioinert systems when developing a SEC method with volatile salts to obtain reliable %HMWs values.

Figure 4B reports the RSD values on elution times. The values were always much lower than the ones obtained for %HMWs. The intraday variabilities on the reference instrument (Waters Acquity UPLC I‐class) and any of the of the other tested systems (Waters Acquity UPLC H‐class Bio and Waters Acquity Premier) were always equal to 0% whatever the analyzed sample. This confirms the excellent repeatability of modern UHPLC instruments and the stability of the prototype SEC column employed in this work. On the other hand, the interday RSD values were equal to only 0.1% for the three different samples. Finally, the interinstrument variability was larger (ranging from 0.1 to 0.2%, depending on the sample) but remains very low, despite the fact that the flow path could have a different volume on the three instruments.

### 3.4 Coupling SEC with MS for size variant identification

#### 3.4.1 Forced degraded study with SEC‐MS

Forced degradation studies are an important part of the development process of therapeutic mAbs and related products [21]. The goal of forced degradation studies is to understand the degradation pathways and establish stability indicating assays to monitor degradation. The conditions of forced degradation studies are often harsh compared to real‐life storage, but helps generate relevant degradation products within a short period of time. For example, degradation under thermal stress is often performed at 35°C or more for several weeks. Since elevated temperature accelerates various degradation pathways (formation of aggregates or fragmentation from peptide bond cleavage), it is one of the most widely explored tests for mAb‐related products.

**FIGURE 5:**
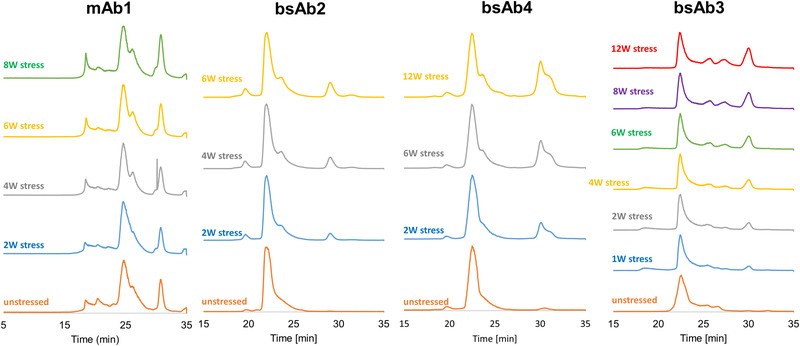
SEC‐MS chromatograms of various unstressed and stressed (40°C for several weeks) mAbs and mAb‐related products. Column: prototype h‐HST SEC. Mobile phase: 50 mM ammonium acetate in water

In the present work, four different biopharmaceutical samples were considered, namely mAb1 (standard mAb), bsAb2, bsAb3, and bsAb4 (three different bispecific samples). These samples were stressed at 40°C for several weeks (up to 6, 8, or 12 weeks depending on the considered sample). Figure 5 shows the corresponding TIC chromatograms obtained for unstressed samples (bottom traces) and various stressed samples under SEC‐MS conditions using the prototype h‐HST BEH200 SEC column and the volatile mobile phase described in Section 3.2. For these experiments, the flow rate was reduced to 50 μL/min to maximize MS sensitivity and injection volume was increased to 10 μL.

For the four selected case studies, the proportion of HMWs and LMWs was found to change over time, confirming that aggregation and fragmentation are degradation pathways that take place when the samples are exposed to thermal stress. This was clearly observed for the bsAb2 sample, where the amount of HMWS eluting at about 20 min increased to a very significant degree after 6 weeks of stress at 40°C. On the same sample, the amount of LMWs eluting at slightly less than 30 min also increased across the force degradation time course. For the two bsAb samples, the amount of LMWs was seen to increase much more rapidly than HMWs. Obviously, by looking over the chromatograms reported in Figure 5, one can see there is a clear need to combine SEC with MS to identify species observed during heat stress and related forced degradation studies.

#### 3.4.2 mAb1 as a case study

**FIGURE 6:**
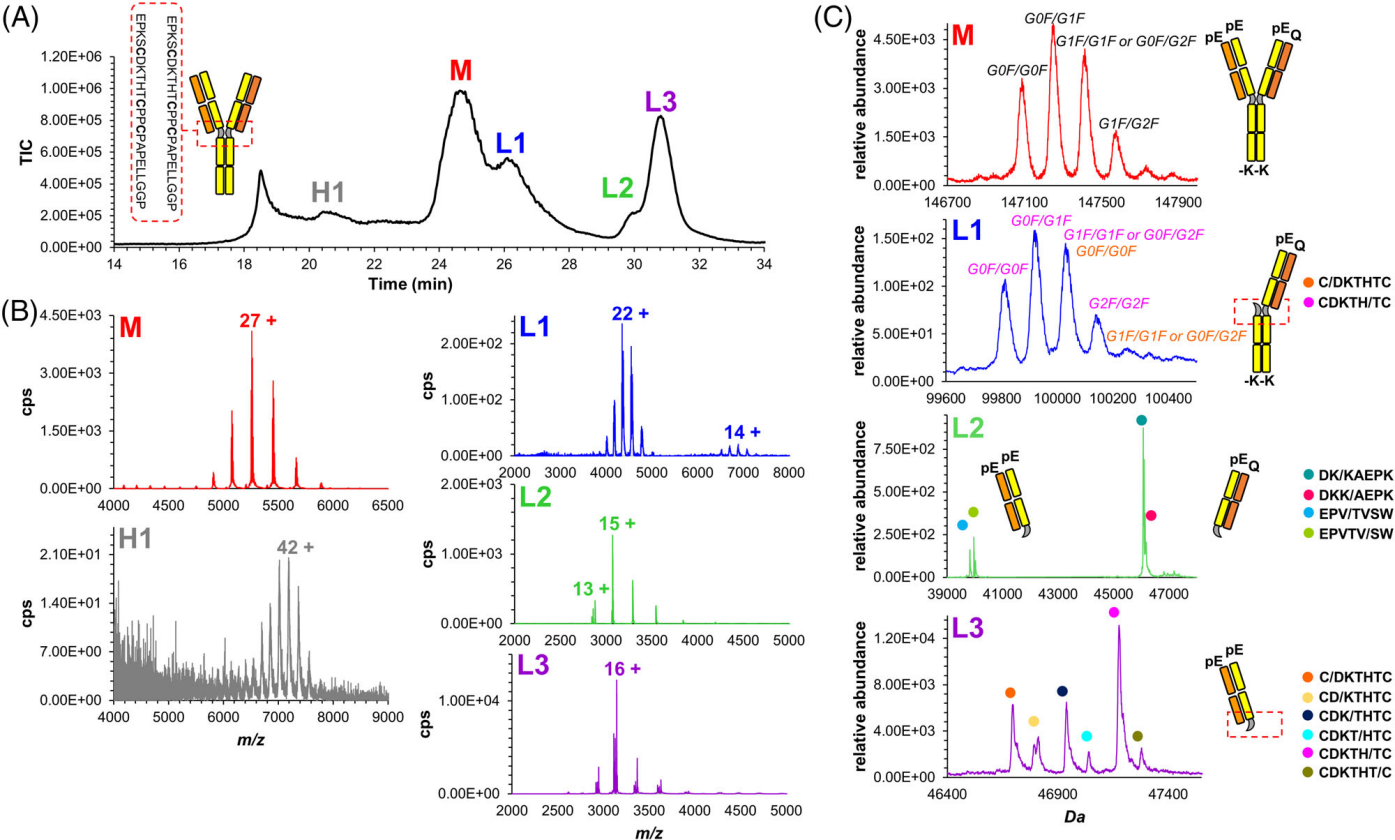
Identification and assignment of size variants observed for stressed mAb1 sample (8 weeks at 40°C) by SEC‐MS. Column: prototype h‐HST BEH200 SEC. Mobile phase: 50 mM ammonium acetate in water

SEC‐MS size variant analysis was performed on the commercial mAb1 after having performed a thermal stress at 40°C for 8 weeks. As reported in Figure 6, a suitable separation of the main peak (denoted as M) from the HMWs and LMWs was obtained. Table S1 provides a detail of this SEC profile in terms of a full list of elution times and mass assignments. Specifically, the HMWs were identified by MS to be a dimer (labeled as D), the first LMWs to be an Fc‐Fab species (labeled as L1) and the following two LMWs to be Fab fragments (labeled as L2 and L3, respectively). As reported in Figure 6 (red box), the deconvoluted mass spectrum for the monomer (M peak) allowed the identification of the main variant as a monomeric species and some heterogeneity corresponding to three N‐terminal modifications (Q/pE) and the clipping of the C‐terminal lysine residues (denoted as 0K). In addition, four different glycoforms were identified, namely G0F/G0F, G0F/G1F, G1F/G1F (or G0F/G2F), and G1F/G2F. Regarding the LMWs L1 and L3, they could be attributed to fragmentation of the heavy chain hinge region at the SCDKTHTCP sequence (Figure S3), as has been many times observed for mAb products [12, 22]. Specifically, L1 was mainly characterized by the Fc‐Fab fragment with a heavy chain clip at the SCDKTH/TCP bond. In addition, several glycovariants were observed for this specific Fc‐Fab fragment, including G0F/G0F, G0F/G1F, and G1F/G1F (or G0F/G2F) along with a very minor G2F/G2F glycoform. In line with what was observed for the monomeric species, all these glycovariants presented the clipping of the C‐terminal Lys residue and the presence of one Q/pE N‐terminal modification (Table S1). An additional Fc‐Fab proteoform derived from the clipping of the heavy chain at SC/DKTHTCP position was also detected, even though only the glycoform G1F/G1F was observed in this case (Figure 6, orange box). Fab fragments derived from the complementary fragmentation sequence co‐eluted with five other proteoforms and found to correspond to the LMWs L3 (Figure 6, green box). In agreement with the assignment provided for the M and L1 size variants, these species were characterized by the presence of two Q/pE N‐terminal modifications (Table S1). Interestingly, the LMWs L2 was identified as containing two Fab proteoforms derived from unusual fragmentation sites, namely clips at several sites of the VDKKAE and EPVTYSWN sequences (Figure S3). These Fab proteoforms showed the presence of both one and two N‐terminal pyroglutamate (pE) (Figure 6, violet box).

#### 3.4.3 Applicability to a complex bispecific antibody product

SEC‐MS analyses were also carried out on two different noncommercial mAb‐based products, using the optimized LC‐MS conditions discussed in Section 3.4.2. It should be noted that the sequences and expected molecular masses of these two products cannot be disclosed, due to confidentiality reasons. Only their main structural features are reported in Figure S1. As compared to a canonical mAb such as mAb1 (∼145 kDa), bsAb2 and bsAb4 had a mass of approximately ∼165 and ∼200 kDa, respectively. This increase in mass is due to the presence of the third binding site present in the C‐terminal position for both products (Figure S1). Canonical glycosylation profiles and N‐/C‐terminal modifications are expected.

**FIGURE 7:**
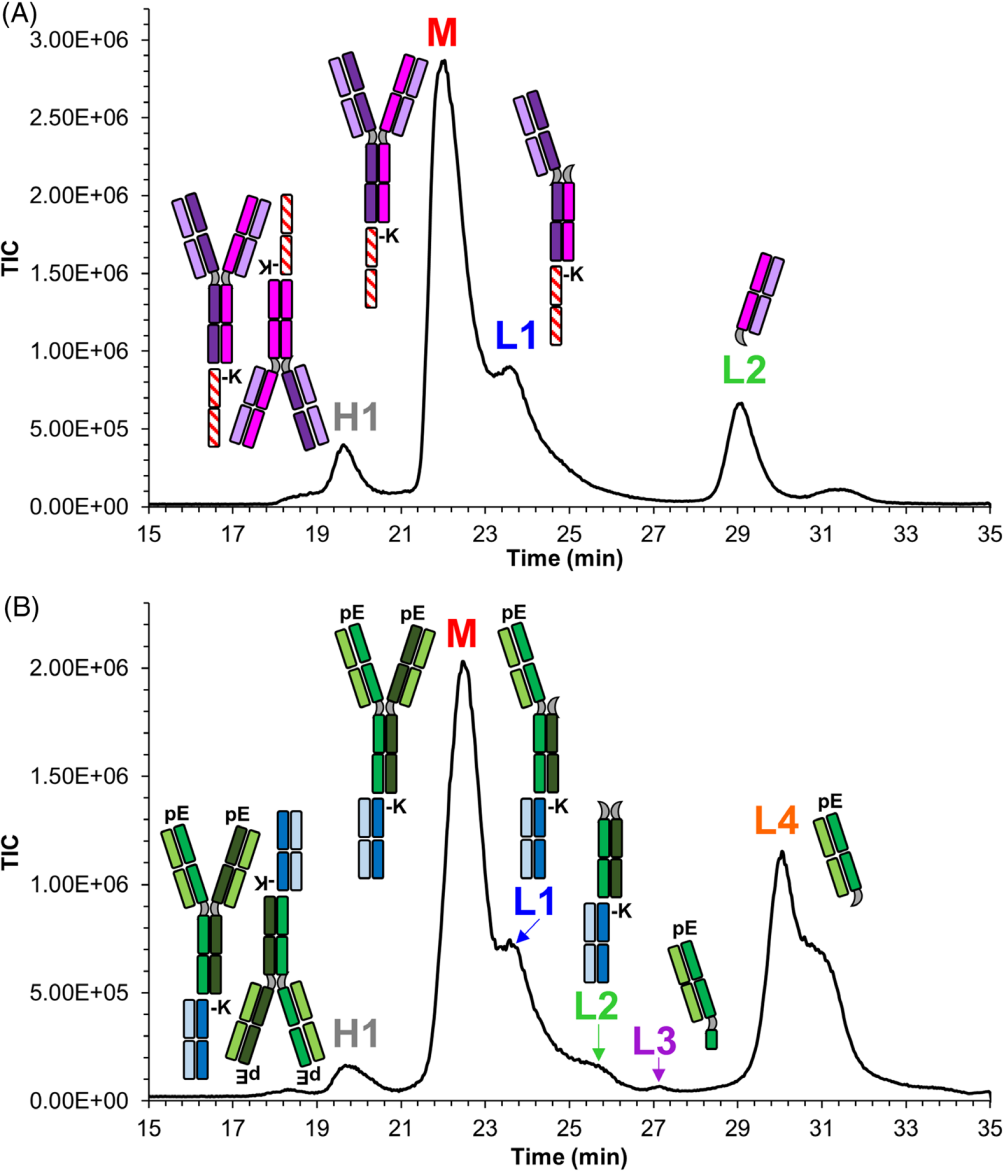
Identification and assignment of size variants observed for stressed (6 weeks at 40°C) bsAb2 sample (A) and stressed (12 weeks at 40°C) bsAb4 sample (B) by SEC‐MS. Column: prototype h‐HST BEH200 SEC. Mobile phase: 50 mM ammonium acetate in water

SEC‐MS analysis of bsAb2 was performed after having applied a thermal stress to the sample at 40°C for 6 weeks. As reported in Figure 7A, the size variant profile consisted of one HMWs (identified as a dimer and labeled as D), the main peak (denoted as M), and two LMWs labeled as L1 and L2. Specifically, L1 was identified as an Fc‐Fab and L2 as the complementary Fab fragment, while the third binding site was never affected by the thermal stress. Glycosylation patterns associated with the M and L1 variants were coherent and consisted of the glycoforms G0F/G0F, G0F/G1F, G1F/G1F (or G0F/G2F), G1F/G2F, and G2F/G2F, all containing the C‐terminal clipping of the lysine residues and no N‐terminal modifications. Both L1 and L2 showed the co‐elution of several (complementary) proteoforms derived from different fragmentation sites (as reported for the mAb1 analysis).

SEC‐MS analysis of bsAb4 was performed after having applied a thermal stress to the sample at 40°C for 12 weeks. As reported in Figure 7B, the size variant profile reveals the presence of one HMWs (identified as a dimer, D), a main species (identified as a monomer, M), and four LMWs labeled as L1–L4. Modifications associated with M included the clipping of the C‐terminal lysine residues and the presence of two N‐terminal pyroglutamates (pE), accompanied by five main glycoforms consisting of G0F/G0F, G0F/G1F, G1F/G1F (or G0F/G2F), G1F/G2F, and G2F/G2F. The same pattern of modifications was found in the LMWs L1, which consisted of an Fc‐Fab variant. Several proteoforms derived from different fragmentation sites were observed for this Fc‐Fab variant. Interestingly, the complementary fragmentation sequences were identified as Fab proteoforms and corresponded to the LMWs L4. The additional LMWs (L2, L3) were quite difficult to assign since they showed noncanonical fragmentation sites. Specifically, L2 was identified as an Fc fragment (still bearing the third binding site at its C‐terminal position), and L3 as a larger Fab fragment as compared to L4, denoting a different fragmentation site of the heavy chains.

Of note, the addition of MS to the SEC analysis was critical to the dentification of all the size variants (especially the LMWs) and for confirming dimerization and assembly of the bsAb monomeric species. In addition, SEC‐MS analysis made it possible to confirm that there were no homo‐dimeric species, one of the main critical analytical challenges in the analysis of bsAbs.

## 4 CONCLUDING REMARKS

This work describes the potential of a novel SEC column with attributes that lend to the analysis of complex protein samples using volatile mobile phase and hyphenated MS detection.

First, we have compared the performance of a reference SS SEC column with that of a prototype version containing the same batch of packing material but a different column hardware surface. The column hardware was prepared with a newly developed hydrophilic HST surface (h‐HST), composed of hybrid organic−inorganic siloxane modified with a hydrophilic layer. To make progress on improving the MS compatibility of SEC, it indeed appears that column hardware material has been as important to address as the chemistry of the packing material. In this case, it is ideal for nondesired chemical interactions (both hydrophobic and electrostatic) to be as minimal as possible. Thanks to the use of this prototype h‐HST SEC column, band broadening, peak tailing, and recovery of HMWs were significantly improved for various complex mAb‐related products when using a volatile mobile phase.

Second, various mobile phases (phosphate and ammonium acetate of differing ionic strengths) were evaluated in combination with the innovative SEC column. It appears that the best compromise between LC performance and MS sensitivity was obtained with an aqueous mobile phase composed of only 50 mM ammonium acetate. Method repeatability was evaluated for these conditions, and excellent intra‐ and interday repeatability was observed for elution times and %HMWs. Larger RSD values were observed for %HMWs when comparing different UHPLC instruments, thus confirming the need to preferentially use bioinert (low adsorption) LC systems.

Finally, the optimized SEC conditions were combined with MS and applied for the size variants characterization of various stressed and nonstressed complex mAb products (canonical mAb and bsAb). The combination of SEC with MS was found to be particularly valuable to the confirmation of proper dimerization and assembly of a bsAb therapeutic and to check the presence/absence of homo‐dimeric species.

## CONFLICT OF INTEREST

Szabolcs Fekete and Matthew Lauvber are employees of Waters (Milford, MA, USA) who have produced the prototype column employed in this work.

## Supporting information

SUPPORTING INFORMATION

## Data Availability

The data that support the findings of this study are available from the corresponding author upon reasonable request.
