# Supplementary material for: Direct coupling of size exclusion chromatography and mass spectrometry for the characterization of complex monoclonal antibody products
Source: J Sep Sci. 2022 Mar 28;45(12):1997–2007. doi: 10.1002/jssc.202200075 (PMC9311719; doi:10.1002/jssc.202200075)
Supplement: Supplementary file 1 — SUPPORTING INFORMATION [file JSSC-45-1997-s001.docx]

**Supporting Information**

**Direct coupling of size exclusion chromatography and mass spectrometry for the characterization of complex mAb products**

**Figure S1**

Cartoon representations of the structures of the various mAbs and related products used in this study


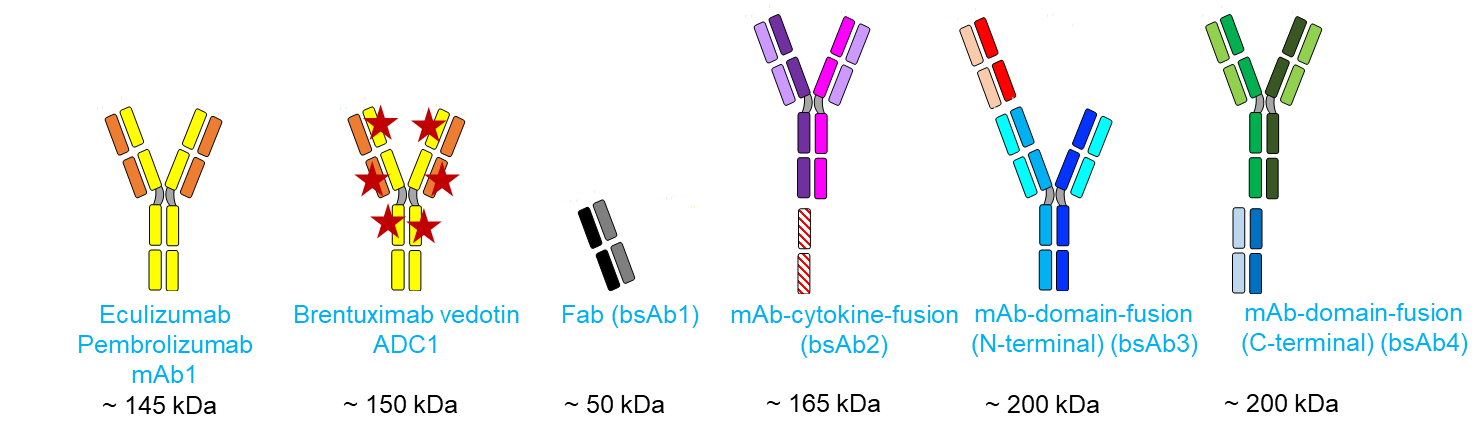


**Figure S2**

Chromatograms obtained with various mAbs and mAb related products under SEC conditions with the reference phosphate mobile phase (50 mM potassium phosphate + 250 mM KCl) and 50 mM ammonium acetate on the prototype Waters BEH200 h-HST SEC column. F = 0.4 mL/min, ACQUITY UPLC I-Class instrument + titanium UV cell.


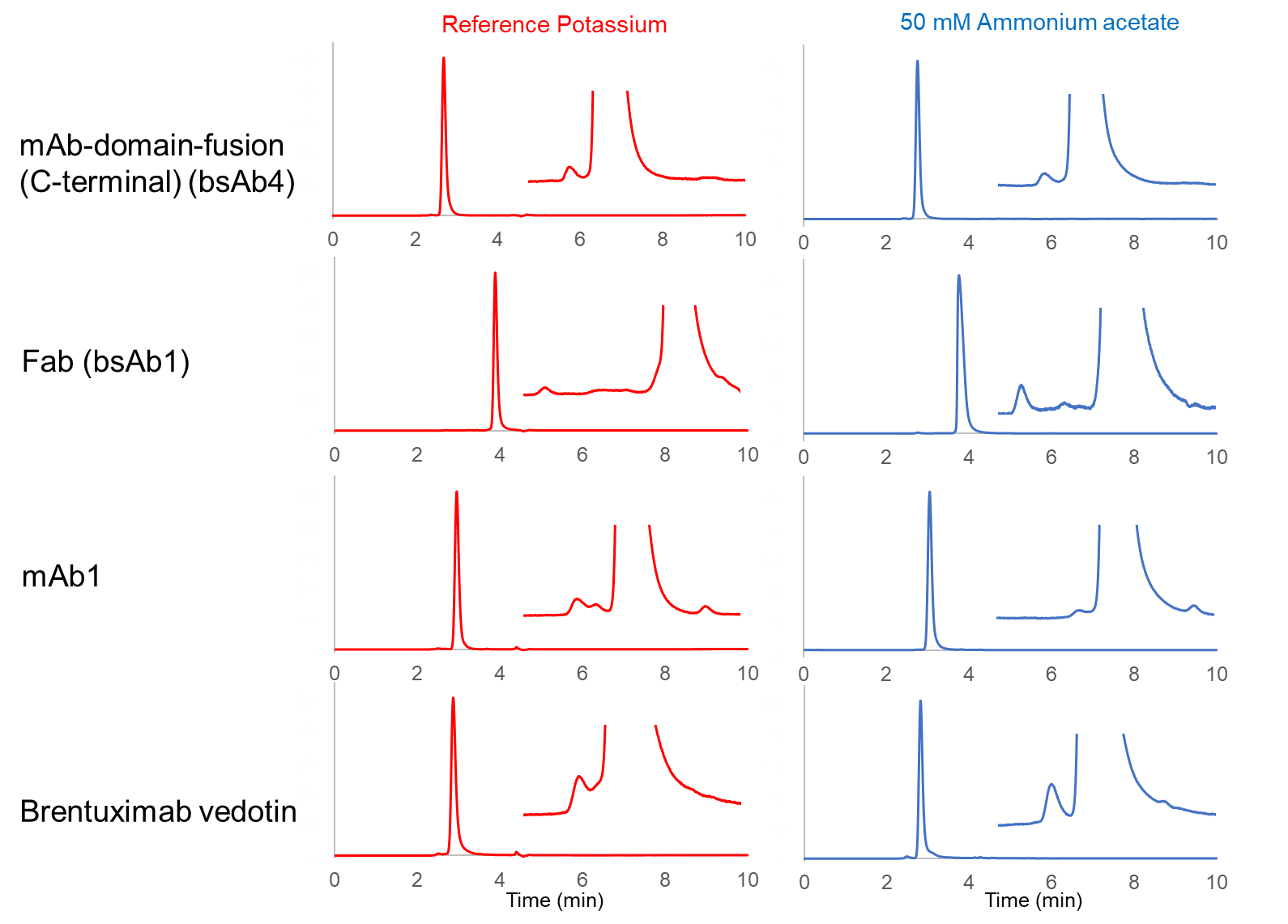


**Figure S3**

Amino acids sequence of mAb1 heavy chain and light chain. N-terminal Glutamine residue ongoing to cyclization (Q/pE) are highlighted in green, the C-terminal Lysine (K) in red, the conserved N-glycosylation site (NST) in pink, and the canonical fragmentation sequence in yellow. In addition, the two non-canonical fragmentation sequences have been highlighted in cyan.

Sequence source: <https://go.drugbank.com/> (last access on 22 Dec 2022)

Heavy chain

QVQLQQPGAE LVKPGASVKM SCKASGYTFT SYNMHWVKQT PGRGLEWIGA IYPGNGDTSY 60

NQKFKGKATL TADKSSSTAY MQLSSLTSED SAVYYCARST YYGGDWYFNV WGAGTTVTVS 120

AASTKGPSVF PLAPSSKSTS GGTAALGCLV KDYFPEPVTV SWNSGALTSG VHTFPAVLQS 180

SGLYSLSSVV TVPSSSLGTQ TYICNVNHKP SNTKVDKKAE PKSCDKTHTC PPCPAPELLG 240

GPSVFLFPPK PKDTLMISRT PEVTCVVVDV SHEDPEVKFN WYVDGVEVHN AKTKPREEQY 300

NSTYRVVSVL TVLHQDWLNG KEYKCKVSNK ALPAPIEKTI SKAKGQPREP QVYTLPPSRD 360

ELTKNQVSLT CLVKGFYPSD IAVEWESNGQ PENNYKTTPP VLDSDGSFFL YSKLTVDKSR 420

WQQGNVFSCS VMHEALHNHY TQKSLSLSPG K 451

Light chain

QIVLSQSPAI LSASPGEKVT MTCRASSSVS YIHWFQQKPG SSPKPWIYAT SNLASGVPVR 60

FSGSGSGTSY SLTISRVEAE DAATYYCQQW TSNPPTFGGG TKLEIKRTVA APSVFIFPPS 120

DEQLKSGTAS VVCLLNNFYP REAKVQWKVD NALQSGNSQE SVTEQDSKDS TYSLSSTLTL 180

SKADYEKHKV YACEVTHQGL SSPVTKSFNR GEC 213

**Table S1.** SEC-MS analysis of mAb1 after a thermal stressed performed for 8 weeks at 40°C. elution times and mass assignment of the different size variants. Of note, the heavy chain (HC) upper hinge region generally subjected to cleavage consists of the following amino acid sequence: SCDKTHTCP, as reported in Figure S3. Analysis was performed with the prototype Waters BEH200 h-HST SEC column.

| **Elution time (min)** | **Peak label** | **Assignment** | **Theoretical mass (Da)** | **Experimental mass (Da)** | **∆m (Da)** | **ppm error** |
| --- | --- | --- | --- | --- | --- | --- |
| 20.65 | D | Dimer | n.a | 294881.88 | n.a | n.a |
| 24.63 | M | Monomer 0K-3Q/pE-G0F/G0F | 147093.68 | 147090.67 | 3.01 | 20 |
|  |  | Monomer 0K-3Q/pE-G0F/G1F | 147255.82 | 147253.75 | 2.07 | 14 |
|  |  | Monomer 0K-3Q/pE-G1F/G1F or G0F/G2F | 147417.96 | 147416.02 | 1.94 | 13 |
|  |  | Monomer 0K-3Q/pE-G1F/G2F | 147580.10 | 147578.59 | 1.51 | 10 |
| 26.10 | L1 | Fc-Fab 0K-1Q/pE (LC+HC+HC clipped at CDKTH/TC from N-terminal) G0F/G0F | 99932.98 | 99930.55 | 2.43 | 24 |
|  |  | Fc-Fab 0K-1Q/pE (LC+HC+HC clipped at CDKTH/TC from N-terminal) G0F/G1F | 100095.12 | 100089.56 | 5.56 | 56 |
|  |  | Fc-Fab 0K-1Q/pE (LC+HC+HC clipped at CDKTH/TC from N-terminal) G1F/G1F or G0F/G2F | 100257.26 | 100251.36 | 5.90 | 59 |
|  |  | Fc-Fab 0K-1Q/pE LC+HC+HC clipped at C/DKTHTC from N-terminal) G0F/G0F | 100414.49 | 100410.49 | 4.00 | 40 |
|  |  | Fc-Fab 0K-1Q/pE (LC+HC+HC clipped at CDKTH/TC from N-terminal) G2F/G2F | 100581.54 | 100584.12 | 2.58 | 26 |
|  |  | Fc-Fab 0K-1Q/pE (LC+HC+HC clipped at C/DKTHTC from N-terminal) G0F/G2F or G1F/G1F | 100738.77 | 100748.7 | 9.93 | 99 |
|  |  | Fab 1Q/pE (LC+HC clipped at VDKK/AEPKSCDKTHTC from  C-terminal) | 46100.54 | 46099.2 | 1.34 | 29 |
|  |  | Fab 2Q/pE (LC+HC clipped at EPV/TVSWN from C-terminal) | 39819.47 | 39819.21 | 0.26 | 7 |
|  |  | Fab 2Q/pE (LC+HC clipped at EPVTV/SWN from C-terminal) | 40019.71 | 40018.58 | 1.13 | 28 |
| 30.81 | L3 | Fab 2Q/pE (LC+HC clipped at C/DKTHTC from C-terminal) | 46697.20 | 46697.14 | 0.06 | 1 |
|  |  | Fab 2Q/pE (LC+HC clipped at CD/KTHTC from C-terminal) | 46812.29 | 46810.95 | 1.34 | 29 |
|  |  | Fab 2Q/pE (LC+HC clipped at CDK/THTC from C-terminal) | 46940.46 | 46940.60 | 0.14 | 3 |
|  |  | Fab 2Q/pE (LC+HC clipped at CDKT/HTC from C-terminal) | 47041.57 | 47040.73 | 0.84 | 18 |
|  |  | Fab 2Q/pE (LC+HC clipped at CDKTH/TC from C-terminal) | 47178.71 | 47179.21 | 0.50 | 11 |
|  |  | Fab 2Q/pE (LC+HC clipped at CDKTHT/C from C-terminal) | 47279.82 | 47278.55 | 1.27 | 27 |
